# Nontuberculous Mycobacteria Lymphadenitis: A Case Report

**DOI:** 10.7759/cureus.846

**Published:** 2016-10-25

**Authors:** Amanda M Krantz, Meera Varnam, Cristina Fernandez

**Affiliations:** 1 School of Medicine, Creighton University; 2 Pediatrics, Creighton University Medical Center

**Keywords:** nontuberculous mycobacterium, ppd

## Abstract

Atypical mycobacteria, also known as nontuberculous mycobacteria (NTM) includes acid-fast bacteria other than *Mycobacterium tuberculosis.* NTM can be isolated from a variety of environmental sources including water, food products, domestic animals, and soil; human exposure is typically from soil to the oral cavity and respiratory tract. Diagnosis of NTM is suspected in children less than five years old with subacute, unilateral, non-tender cervicofacial lymphadenitis in combination with a history of water exposure, penetrating injection, as well as negative routine cultures or response to antistaphylococcal and antistreptococcal antibiotics. The course of the disease is variable and can involve eruption of the lymph node and tract formation with drainage. Management of nontuberculous mycobacteria can include surgical and antimycobacterial therapy. We present a case of a two-year-old African American girl who presented to the clinic with anterior ear lobe and submandibular lymphadenitis due to suspected NTM.

## Introduction

Atypical mycobacteria, also known as nontuberculous mycobacteria (NTM), includes acid-fast bacteria other than *Mycobacterium tuberculosis *(MTB). There are four categories of NTM: photochromogens, scotochromogens, nonphotochromogens, and fast growers. NTM can be isolated from a variety of environmental sources, including water, food products, domestic animals, and soil. Exposure of the human oral cavity and respiratory tract to NTM is typically from soil [1-3]. Incubation periods are variable but can reach up to five years. Infected pediatric patients are immunocompetent, and race and gender do not seem to play a predisposing role in the development of NTM in the pediatric population [4]. The prevalence of NTM seems to be increasing, though this may be a result of enhanced detection [5]. Informed consent was obtained from the patient's parent/guardian for this study.

## Case presentation

A two-year-old African American girl presented to the clinic with anterior ear lobe and submandibular lymphadenitis. Incision and drainage were previously performed in the emergency room one month before this visit. When a suppurative node persisted, further evaluation in the clinic was performed due to suspicion of chronic infection. Antibody titers against Ebstein-Barr virus (EBV) and Cytomegalovirus (CMV) were assayed (Table 1) to rule out viral etiologies. A chest radiograph was taken and found to be negative, ruling out a primary pulmonary source of tuberculosis. A positive purified protein derivative (PPD) test was also performed and found to measure 12 millimeters. This was interpreted as positive due to our patient's age of less than four years per the United States Centers for Disease Control (CDC) guidelines. 

**Table 1:** Laboratory Studies Laboratory evaluations including CMV and EBV results

Lab Study	Result	Reference	Inference
CMV Ab IgG	4.10U/ml	0.7 U/ml >	Prior Exposure
CMV Ab IgM	<8.0AU/mL	< 29.9 AU/mL	Negative Result
EBV Ab to Viral Capsid Ag IgG	411.0U/ml	22.0 U/mL >	Prior Exposure
EBV Ab to Viral Capsid Ag IgM	<10.0U/mL	< 35.9 U/mL	Negative Result
EBV Ab to Nuclear Ag IgG	460.0U/mL	22.0 U/mL >	Prior Exposure

The follow-up visits at four and eight weeks after initiation of therapy revealed a resolution of the lymphadenitis, without recurrence. Subsequent follow-up visits were scheduled at eight week intervals to monitor for return of the lesion and adverse side effects to antibiotic therapy.

## Discussion

Lymphadenopathy in the pediatric population is not uncommon, and its etiology stems from a variety of both infection and non-infectious agents. While generalized lymphadenopathy is more commonly associated with a systemic disease process, peripheral lymphadenopathy is typically associated with an infectious etiology. Other differential diagnoses for a patient with localized peripheral lymphadenopathy include granulomatous disease, neoplasia, or reactive hyperplasia of unknown etiology. Considerations should be given to the patient's age, the length of lymphadenopathy, and the presence of alarm symptoms [6-7].

A diagnosis of NTM lymphadenitis should be suspected in children less than five years old with subacute, unilateral, non-tender cervicofacial lymphadenitis that improves with appropriate antibiotic therapy. Submental and anterior cervical lymph nodes are routinely involved. Lymphadenitis, in combination with a history of water exposure or penetrating injection, as well as negative routine cultures or response to antistaphylococcal and antistreptococcal antibiotics, should increase clinical suspicion of an NTM infection [1]. Pediatric patients are commonly infected after putting wet dirt or soil into their mouths. 

Diagnosis is made by acid-fast staining, mycobacterial culture, and histopathology. Histopathologic features can include sinus tracts, inflammation, and dermal granulomas. A positive culture will confirm the diagnosis but can take up to six weeks [3]. The growth characteristics in media distinguish NTM into two general categories: slowly-growing (*M. fortuitum, M. chelonei, and M. abscessus*) and rapidly-growing mycobacteria (*M. marinum, M. Kansasii, and M. avium-intracellulare*) [4]. In the United States, the majority of cases of NTM are associated with *M. avium complex* [2]. Speciation is imperative to ensure appropriate antimycobacterial treatment. Differential diagnosis in immunocompromised patients for NTM includes MTB (*M. Tuberculosis),* nocardiosis (*Nocardia asteroides*), sporotrichosis (*Sporothrix schenckii*), and cutaneous leishmaniasis [4]. A PPD skin test may be positive, though the test cannot distinguish between MTB and NTM. Though false positive PPD results can occur without infection of nontuberculous mycobacterium after vaccination with the Bacillus Calmette–Guérin (BCG) vaccine, our patient had not been vaccinated with the BCG vaccine. 

As cervicofacial lymphadenopathy can be caused by viral etiologies, antibody assays can be performed to evaluate the patient's exposure to CMV or EBV. In our case, the positive CMB Ab IgG result indicates a previous exposure to CMV, but as the CMV Ab IgM was negative, the patient did not have an active CMV infection. A positive viral capsid IgG and positive nuclear IgG indicate a past EBV infection. 

Lymph node biopsy or incision and drainage are contraindicated in suspected NTM lymphadenitis if the etiology is clear and if the lymphadenopathy is expected to improve with no further management. A relative contraindication to biopsy is recognized if the suspected etiology can be treated expectantly (e.g. with administration of antibiotic). In our case, the latter held true, as the patient improved after appropriate antibiotics were initiated.

The course of NTM is variable and can involve eruption of the lymph node and tract formation with drainage; the lymph node can also remain indurated [1]. Systemic symptoms are unusual in immunocompetent patients. In patients with underlying conditions, including pulmonary disorders and immune compromise, there is an increased risk of pulmonary disease and disseminated disease respectively [2]. Reactivation of NTM can occur after trauma or injury near the affected area [3].

Management of NTM can include surgical and/or antimycobacterial therapy. Empiric therapy of NTM should include macrolide (azithromycin or clarithromycin), fluoroquinolone, or trimethoprim-sulfamethoxazole. Follow-up visits to monitor response to therapy are suggested and therapy is indicated for one year [2]. If the patient does not improve on specific therapy after four to six weeks, the regimen can be modified [6]. Patients on long-term antibiotics should be monitored for adverse effects including nausea, weight loss, erythema, and peripheral eosinophilia [1]. NTM complications include penetration into deeper tissues, inoculation of muscle and bones, and a resulting local infection; other significant complications that should be monitored for include tenosynovitis, osteomyelitis, and pyomyositis [3].

## Conclusions

In the pediatric population, lymphadenitis is not uncommon, and NTM is a less common etiology of cervical lymphadenitis. NTM exposure to the oral cavity of pediatric patients is frequently through soil. In patients less than five years old with facial lymphadenitis and a history of exposure, evaluation for NTM should be considered. Without appropriate management, a suppurative node or tract formation can persist. While culture and histopathology can be used to make a definitive diagnosis, results can take up to six weeks, and empiric management with antibiotics is appropriate in situations where the patient is expected to improve on therapy. Intervention with empiric antibiotic therapy should be initiated for 12 months, and the drug choice can be modified once susceptibility has been established.
